# *Solenostemon monostachyus*, *Ipomoea involucrata* and *Carica papaya* seed oil versus Glutathione, or *Vernonia amygdalina*: Methanolic extracts of novel plants for the management of sickle cell anemia disease

**DOI:** 10.1186/1472-6882-12-262

**Published:** 2012-12-22

**Authors:** Israel Sunmola Afolabi, Iyanuoluwa Olubukola Osikoya, Oluwabukunmi Dorcas Fajimi, Priscilla Ibanga Usoro, Damilola Olufunlayo Ogunleye, Tolulope Bisi-Adeniyi, Alaba O Adeyemi, Bosede Temitope Adekeye

**Affiliations:** 1 Biological Sciences Department, Covenant University, College of Science and Technology, Biochemistry Unit, Canaan Land, Km. 10, Idiroko Road, P.M.B. 1023, Ota, Ogun State, Nigeria

**Keywords:** Sickle cell disease, Health, Management, Antisickling, Underutilized, Plants

## Abstract

**Background:**

Sickle cell disease (SCD) is a genetic disease caused by an individual inheriting an allele for sickle cell hemoglobin from both parents and is associated with unusually large numbers of immature blood cells, containing many long, thin, crescent-shaped erythrocytes. It is a disease prevalent throughout many populations. The use of medicinal plants and nutrition in managing SCD is gaining increasing attention.

**Methods:**

The antisickling effects of *Solenostemon monostachyus* (SolMon), *Carica papaya* seed oil (Cari-oil) and *Ipomoea involucrata* (Ipocrata) in male (HbSSM) and female (HbSSF) human sickle cell blood was examined *in vitro* and compared with controls, or cells treated with glutathione or an antisickling plant (*Vernonia amygdalina*; VerMyg).

**Results:**

Levels of sickle blood cells were significantly reduced (P < 0.05) in all the plant-extract treated SCD patients’ blood compared with that of untreated SCD patients. RBCs in SolMon, Ipocrata, and Cari-oil treated samples were significantly higher (P < 0.05) compared with VerMyg-treated samples. The Fe^2+^/Fe^3+^ ratio was significantly reduced (P < 0.05) in all plant extract-treated HbSSM samples compared with controls. Hemoglobin concentration was significantly increased (P < 0.05) by SolMon treatment in HbSSF compared with VerMyg. Sickle cell polymerization inhibition exhibited by SolMon was significantly higher (P < 0.05) compared with that of VerMyg in HbSSF blood. Sickle cell polymerization inhibition in SolMon and Ipocrata were significantly higher (P < 0.05) compared with VerMyg in HbSSM blood. All plant extracts significantly reduced (P < 0.05) lactate dehydrogenase activity in both HbSSM and HbSSF-treated blood. Catalase activity was significantly increased (P < 0.05) in HbSSF blood treated with Ipocrata compared with glutathione. Cari-oil treated HbSSM and HbSSF blood had significantly increased (P < 0.05) peroxidase activity compared with controls.

**Conclusions:**

Methanolic extracts from *S*. *monostachyus*, *C*. *papaya* seed oil and *I*. *involucrata* exhibited particular antisickling properties coupled with the potential to reduce stress in sickle cell patients. Each plant individually or in combination may be useful for the management of sickle cell disease.

## Background

The practice of traditional medicine using medicinal plants has a long history in many cultures. This type of health care can be described as herbalism or botanical medicine [1]. The growing sophistication in lifestyles among world populations makes it imperative to refer to herbal practice as alternative or complementary medicine, to appeal to a cross section of people irrespective of their cultural affiliation [1]. This type of herbal practice is gaining increasing attention [1]. Two-thirds of the world’s population (mainly in developing countries) relies entirely on such traditional medical therapies as their primary form of health care [2]. The use of traditional medicine is important for the treatment and management of a number of diseases in the African continent [3], as a lack of basic health care and medical personnel make it difficult to treat rural populations.

Sickle cell disease (SCD) is an autosomal recessive genetic disease caused by a mutation in the hemoglobin gene and is associated with shortness of breath, heart palpitations, abdominal pains, and muscular aches and pains [4]. Blood of sickle cell patients contains an unusually large number of immature cells and many long, thin, crescent-shaped erythrocytes that resemble the blade of a sickle [5]. When hemoglobin from sickle cells (called hemoglobin S) is deoxygenated, it becomes insoluble and forms polymers that aggregate to tubular fibers. The altered properties of hemoglobin S result from a single amino acid substitution of valine to glutamate at position 6 in the two hemoglobin chains [5]. The R group of valine has no electric charge, whereas glutamate has a negative charge at pH 7.4. Therefore, hemoglobin S has two fewer negative charges than hemoglobin A, one for each of the two chains. Replacement of the glutamate residue by valine creates a “sticky” hydrophobic contact point at position 6 of the chain, on the outer surface of the molecule. These sticky spots cause deoxyhemoglobin S molecules to associate abnormally with each other, forming long, fibrous aggregates characteristic of this disorder [6]. Increased free intracellular Ca^2+^ occurs during SCD [7], resulting in a loss of K^+^ with accompanying changes in levels of Cl^-^ ions and water, and a corresponding gain of Na^+^. Small blood vessels are blocked by the clumping of sickle red blood cells (RBCs), preventing blood supply to various organs [1].

The anti-sickling effects of various substances and the role of nutrition in overcoming SCD have been reported [8-10], although information on the management of SCD is still scarce. The antioxidant capability of *Solenostemon monostachyus*, *Ipomoea involucrata*, and *Carica papaya* seed oil has recently been reported [11-13]. Some plants and herbs with potent antioxidant activity, including the leaf of *Vernonia amygdalina* and unripe *C*. *papaya* fruit, have been reported to have antisickling properties [14,15]. However, the antisickling properties of *C*. *papaya* seed oil, *I*. *involucrata* and *S*. *monostachyus* leaves have not been reported. This study examined the antisickling effects of aqueous methanolic extracts of leaves from these three plants on human sickle cell blood *in vitro*.

## Methods

### Chemicals

Glucose-6-phosphate dehydrogenase (G6PDH) kit and lactate dehydrogenase (LDH) kit were obtained from Randox Laboratories Ltd. (Crumlin, United Kingdom). Methanol and liquid paraffin were obtained from British Drug House Chemicals Limited (Poole, United Kingdom). Hydrogen peroxide (H_2_O_2_) was obtained from Merck Inc (Lehrte, Germany), and pyrogallol, and glutathione were obtained from Sigma Chemical Incorporation (Munich, Germany). All chemicals used were of analytical grade.

### Collection of plant materials

*S*. *monostachyus* (P. Beauv) Brig. (SoleMon; FHI108913), *I*. *involucrata* P. Beauv. (Ipocrata; FHI 108917), mature ripe *C*. *papaya* fruits (Cari; FHI108906) and *V*. *amygdalina* Del. (VerMyg; FHI 108914) plants were collected between 21^st^ November and 2^nd^ December, 2011 within the lawns of Covenant University, Ota, Ogun State, Nigeria. These plants were identified at the Applied Biology Unit of the Biological Sciences Department at Covenant University. They were reconfirmed and deposited at the Herbarium unit of the Forest Research Institute of Nigeria (FRIN), Ibadan, Nigeria. The allocated voucher numbers for each of the plants deposited at FRIN are as indicated above for each plant. The samples were air dried at ambient temperature (37.8 ± 4.7°C) and turned over regularly to aid in the drying process over a period of 2 weeks. Seeds harvested from the *C*. *papaya* fruits were oven died at 35–37°C for 2 weeks. The dried plants were blended into powdered form using the dry program of a blender (Emel EM-242) and stored in clean air-tight containers prior to use.

### Preparation of methanolic extracts from plants

Preparation of methanolic extracts from plants was carried out using a previously described method with some modifications [15]. Dried samples (90 g) were macerated in 1200 ml of methanol (absolute) for 72 h at room temperature. These were consecutively filtered using cotton wool, a Whatman filter paper (no. 1), and thereafter with a Vane pressure/vacuum pump filtration device (XZ-1B). The collected filtrates were evaporated *in vacuo* using a rotary evaporator (Stuart RE: 300) at 20°C.

### Sample extract concentration

Dried extracts (0.1 g) were re-dissolved in 5 ml of absolute methanol, and filtered using Whatman filter paper (no. 1). The filtrates were then used as the working antisickling agent.

### Collection of blood samples and preparation of serum samples

Sickle cell blood (HbSS) samples from males and females were obtained from General Hospital, Gbagada, Lagos State. The blood samples were collected from confirmed sickle cell patients attending a weekly hematology outpatient clinic at the hospital. Blood samples for healthy males (NBM) and females (NBF) were obtained from the Covenant University Health Center, Canaan Land, Ota, Ogun State, Nigeria. The blood samples were collected from healthy students for this study. The permission of Covenant University Ethical Committee was obtained prior to the commencement of this work. Consent from all blood donors were obtained after they were adequately informed of the research objective. Hospital ethical regulations were observed when blood was collected.

The venipuncture method was used for blood collection as described previously [15]. Blood (4 ml) was drawn from each sickle cell patient (ages 15 to 48 years) at steady state using new syringes and needles, spirit swaps and a tourniquet. The blood samples were collected into EDTA and plain bottles and the collected blood was subsequently inverted gently for mixing. The same procedure was used to collect blood from confirmed normal healthy blood donors selected randomly in Covenant University, except they were 18 to 23 years of age. All experiments were performed within 72 h of blood collection. The blood samples collected were centrifuged at 10 000 rpm for 15 minutes to obtain plasma samples (2.0 ml).

### Packed cell volume (PCV)

Fresh blood samples were collected and dispensed to fill about ¾ of a micro hematocrit tube (75 μL), and plasticine was used to block the opening to avoid spillage during centrifugation at 12 000 rpm for 5 min. PCV was measured using a hematocrit reader.

### Preparation of RBC

One drop of blood obtained after removing plasma was applied to a microscope slide using a Pasteur pipette. The erythrocyte sample was then spread using the tip of the Pasteur pipette. The slides were left to dry, and stained with Leishman’s stain. The slides were kept until used for cell counting.

### Sickle cell reversal

The extent of sickle cell reversal was determined as previously described [15]. HbSS blood (0.2 ml) was placed in a test tube, and 0.2 ml phosphate buffered solution (PBS, 0.05M, pH 8.0) was added. The mixture was covered with liquid paraffin (1.0 ml). 2% sodium metabisulfite solution (0.6 ml) was introduced into the blood layer under the liquid paraffin using a syringe and needle. The mixture was gently mixed before incubating at 37°C in a thermostated water bath for 90 min. At the end of this incubation period, the methanolic extract (0.2 ml) of each plant was added under the liquid paraffin carefully as before and incubated for another 6 h. A 0.2 ml aliquot of PBS and 0.2 ml of blood collected from healthy donors were used in place of the extracts, and HbSS blood respectively to serve as a control. The liquid paraffin layer was carefully removed using a Pasteur pipette and 5% buffered formalin solution (3 ml) was added at the end of the 6-h incubation. The solution was thoroughly mixed to ensure proper fixation and was kept at ambient temperature until ready for counting. The slide was mounted on a camera equipped Lab Biological microscope (Model N-800M) supplied by Tunnex Laboratory Nigeria Limited (Lagos, Nigeria). The number of RBCs and sickle cells were counted to monitor the level of sickling reversal of each plant treatment. The sickling reversal activity for each plant was monitored by calculating their percentage sickle cell blood level using the formula below:

$$\begin{array}{l} \mathrm{Sickle}\;\mathrm{cell}\;\mathrm{blood}\;\mathrm{level}\left(\%\right)=\mathrm{HbSS}\;\mathrm{counted}/\\ \;\left(\mathrm{HbSS}\;\mathrm{counted}+\mathrm{RBC}\;\mathrm{counted}\right)\times 100 \end{array}$$

### Determination of glucose-6-phosphate dehydrogenase (G6PDH) activity

The G6PDH activity was measured according to the manufacturer’s instructions. Fresh blood samples (0.2 ml) were washed with aliquots of 0.9% sodium chloride solution (2 ml) and centrifuged at 300 rpm for 10 min. The washing process was repeated three times. The erythrocyte residue was then suspended in 0.5 ml of digitonin (solution 4 of the G6PDH kit) that had been previously dispensed into an eppendorf tube. After standing for 15 min the suspended erythrocytes were centrifuged again at 300 rpm for 10 min. An aliquot of the hemolysate (15 μl) was added to Reagent R1 buffer (1000 μl), and NADP reagent 2 (30 μl) of the G6PDH kit. The mixture was shaken and incubated at 37°C for 5 min. The initial absorbance was measured at 340 nm immediately after adding the substrate reagent R3 (15 μl). Distilled water was used as a blank. Changes in the absorbance at 340 nm were measured every min for three min. The G6PDH activity was calculated as indicated below following the method described by the kit manufacturer.

$$\begin{array}{l} G6\mathrm{PDH}\;\mathrm{activity}\left(\mathrm{mU}/\mathrm{erythrocytes}\;\mathrm{per}\;\mathrm{ml}\;\mathrm{blood}\right)\\ \;=33650\times \Delta \;\mathrm{absorbance}340\mathrm{nm}/\min \end{array}$$

### Determination of Fe^2+^/Fe^3+^ ratio of sickle cell blood

The effect of the different plant extracts on the Fe^2+^/Fe^3+^ ratios in blood were performed as previously described [8]. An aliquot of sickle cell blood (20 μl) was added to distilled water (5.0 ml) and normal saline (20 μl) to serve as a control. The standard and test solutions contained the same reagents as the control except that an aliquot of the plant extract (20 μl) was added instead of normal saline. Samples were incubated for 60 min and the absorbance measured by spectrophotometer at wavelengths of 540 nm and 630 nm to determine the percent hemoglobin and methemoglobin respectively. The ratio of hemoglobin and methemoglobin was calculated by dividing the percentage hemoglobin by that of methemoglobin.

### Determination of sickle cell polymerization inhibition

Sickle cell polymerization inhibition was carried out as described previously with modifications [8]. Briefly, blood samples from confirmed homozygous SCD patients (HbSS) were hemolyzed by freeze thawing, and subsequently centrifuged at 10 000 rpm for 9 min. The supernatant (hemoglobin) obtained was placed in vials and stored at 4°C. An aliquot of 1.4 ml sodium metabisulfite solution (2% w/v), 80 μl of plant extract (serving as antisickling agent), and HbSS hemoglobin (0.1 ml) were pipetted into a cuvette, shaken and the absorbance measured at 700 nm for 30 min. The same reagents were set up for the controls, except that the plant extract was replaced with the same amount of normal saline (0.5 N). The rates of polymerization were estimated by calculating the rate of change in absorbance per minute.

### Determination of lactate dehydrogenase (LDH) activity

This was outperformed according to the manufacturer’s instructions. An aliquot of the plant extract (20 μl) was dispensed into a cuvette containing the kit reagent (1.0 ml), and serum samples (40 μl) of the HbSS patients. Serum samples from healthy contributors were used for the control. All the reactions were carried out at 25°C. The initial absorbance at 340 nm was taken within 30 s, and the absorbance changes were taken afterwards at every 1 min for 3 min. The LDH activity was calculated using the formula below as provided by the LDH kit manufacturer.

$$\begin{array}{l} \mathrm{LDH}\;\mathrm{activity}\left(U/I\right)=4127\\ \;\times \Delta \;\mathrm{Absorbance}\;340\;\mathrm{nm}/\min \end{array}$$

### Determination of catalase activity

Catalase activity of plant extracts on human blood was assayed as previously described with modifications [16]. Briefly, an aliquot of hydrogen peroxide (0.8 ml) was dispensed into an eppendorf tube. Phosphate buffer (1.0 ml), extracted sample (75 μl) and (0.002% v/v) diluted blood (125 μl) were added. The reaction mixture (0.5 ml) was dispensed into 5% dichromate reagent (1.0 ml) and vigorously shaken. The mixture was heated in a Clifton water bath for ten min, and allowed to cool. The initial absorbance at 570 nm was taken within 10 s of adding the substrate, and at 60 s and 120 s using a Surgispec spectrophotometer (SM-23A). Glutathione was used as a positive control for the tests. The absorbances obtained were extrapolated from a prepared standard graph. The catalase activity was thereafter expressed as μmole/min using the following formula:

$$\begin{array}{l} \mathrm{Activity}\left(\mu \mathrm{mole}/\min\right)=\Delta \;\mathrm{optical}\;\mathrm{density}\\ \;\times H_{2}0_{2}\mathrm{standard}\;\mathrm{concentration}\left(\mu \mathrm{mol}\right)\\ \;/\mathrm{enzyme}\;\mathrm{volume}\left(\mathrm{ml}\right)\times \mathrm{standard}\;\mathrm{optical}\;\mathrm{density} \end{array}$$

### Determination of peroxidase activity

Peroxidase activity was determined as previously described [17]. An aliquot of PBS (0.1 ml), hydrogen peroxide (50 μl), and pyrogallol solution (110 μl) were added to distilled water (625 μl) that was earlier dispensed into an eppendorf tube. The plant extract (75 μl) was thereafter added. The same reagents were used, except the extract was replaced by distilled water (75 μl), for the blank. The reaction was mixed and incubated for at least 10 min. The solution containing 100 mM, pH 6.0 PBS (40 μl) and 0.002% (v/v) diluted blood sample (40 μl) were added to the blank and test mixtures respectively. These were mixed, and the increase in absorbance at 420 nm was measured at every 10 s for 3 min using a Surgispec spectrophotometer (SM-23A). Glutathione was used as a positive control for the tests. One unit of peroxidase was defined as the change in absorbance/seconds at 420 nm.

### Statistical analysis

One factor randomized complete block design was used for this experiment. Data were analyzed using analysis of variance (ANOVA) MegaStat statistical software package supplied by Dataville Solutions Ltd. (Lagos, Nigeria). Treatments with a P value of < 0.05 were considered statistically significant and were subjected to a Student *t*-test using the same statistical software. The results were expressed as mean ± standard deviation.

## Results

### Effect of plant methanolic extract-treatment on PVC

The PCV in NBM was significantly higher (P < 0.05) compared with that of NBF blood donors. The PCV in HbSSM or HbSSF was significantly lower (P < 0.05) compared with either NBM or NBF blood (Table 1). Sickle blood cell counts were significantly reduced (P < 0.05) in all the plant-extract treated SCD patients’ blood compared with that of untreated SCD patients’ blood. Plant extract-treated sickle cell blood had significantly higher (P < 0.05) numbers of sickle blood compared with that of healthy blood donors. However, there was no significant difference (P > 0.05) in the number of sickle RBCs for all plant extract-treated groups compared with VerMyg (Table 2). Only the Ipocrata-treated group had a significantly higher (P<0.05) percentage of sickle cell blood compared with that of the *V*. *amygdalina* group.

**Table 1:** Clinical features of blood collected from sickle cell patients and healthy donors

**Parameters**	**Healthy Blood**		**Sickle Blood**	
	**Male**	**Female**	**Male**	**Female**
PCV (%)	43.20 ± 2.74	32.90 ± 3.28	22.70 ± 8.27	23.90 ± 5.76
G6PDH (mU/ml)	55.81 ± 16.24	56.71 ± 13.94	147.30 ± 16.20	69.34 ± 12.88

Data expressed as mean ± SD (*n* = 10).

G6PDH = glucose-6-phosphate dehydrogenase; PCV = packed cell volume.

**Table 2:** Blood counts in healthy donors and sickle cell patients treated with some plant extracts

**Sample**	**Blood count**		**Sickle cell level (%)**
	**No. of Sickle cell RBC**	**No. of RBC per 10 field**	
Control (healthy donor)	0.00 ± 0.00	958.00 ± 10.95	0.00
Control (untreated HbSS)	246.20 ± 30.77	670.00 ± 23.91	26.85 ± 3.12
*Vernonia amygdalina*	20.00 ± 10.27	654.40 ± 98.62	2.42 ± 1.11
*Ipomoea involucrate*	33.60 ± 9.89	864.60 ± 46.17	4.04 ± 0.71
*Solenostemon monostachyus*	18.40 ± 4.16	851.00 ± 111.66	1.90 ± 0.43
*Carica papaya* seed-oil	12.40 ± 6.11	961.40 ± 157.70	1.03 ± 0.16

Data expressed as mean ± SD (*n* = 5). RBC = red blood cell. HbSS = human sickle cell blood.

**Figure 1:**
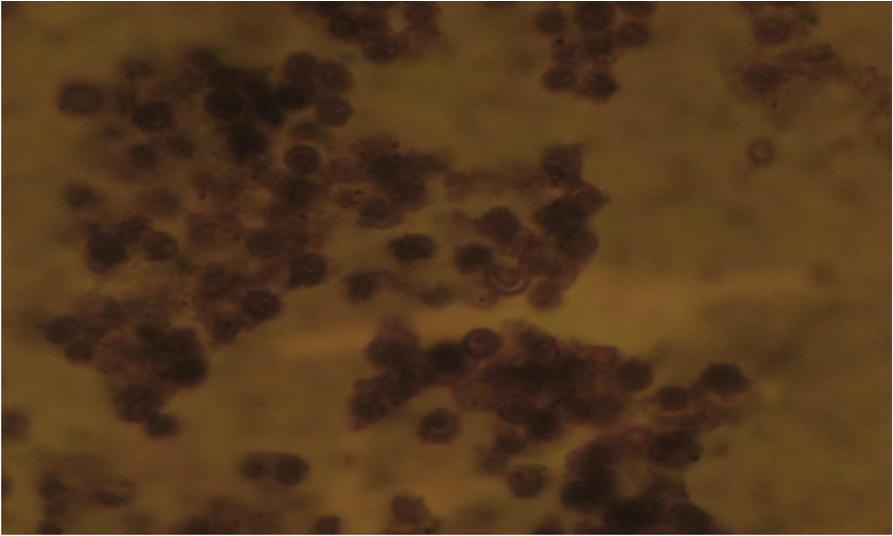
Picture of blood collected from a sickle cell blood (Magnification: x1000).

**Figure 2:**
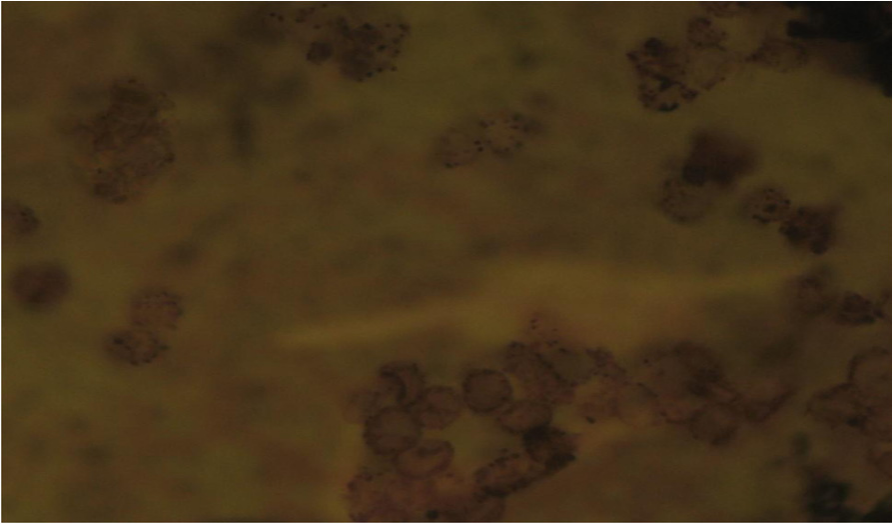
**Picture of sickle cell patient blood treated with *Ipomoea involucrata *(Magnification: x1000).**

**Figure 3:**
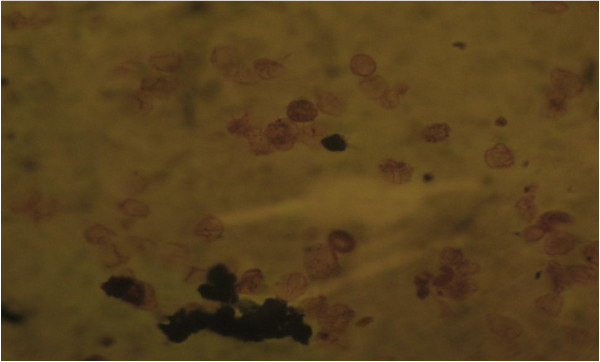
**Picture of sickle cell patient blood treated with *Solenostermon monostachyus *(Magnification: x1000).**

**Figure 4:**
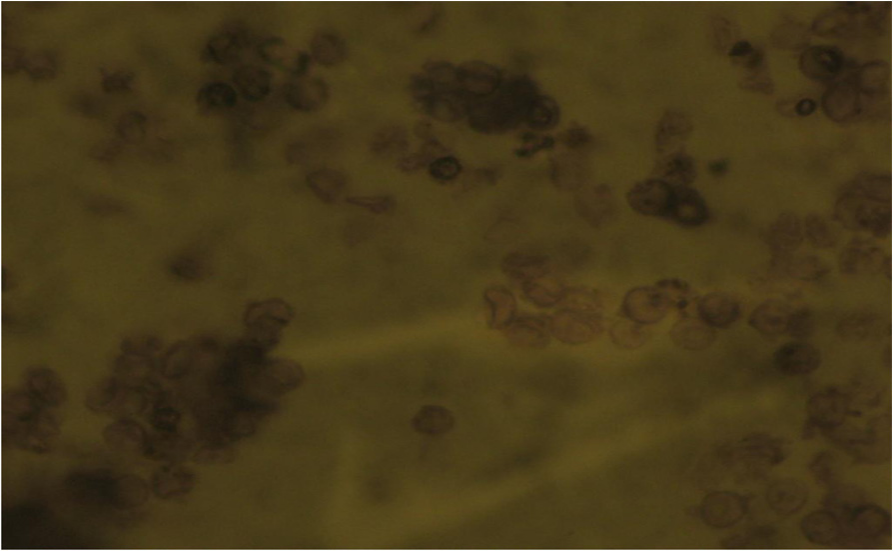
**Picture of sickle cell patient blood treated with *Carica papaya *seed oil (Magnification: x1000).**

**Figure 5:**
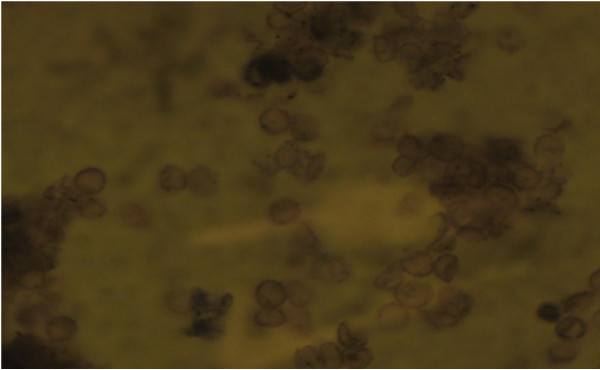
**Picture of sickle cell patient blood treated with *Vernonia amygdalina *(Magnification: x1000).**

The number of RBCs per 10 fields of view were significantly lower (P < 0.05) in the VerMyg and Ipocrata extract-treated sickle blood compared with that of healthy blood donors, whereas the number of RBCs per 10 fields of view in SolMon-, Ipocrata- and Cari-oil-treated groups were significantly higher (P < 0.05) compared with that of VerMyg (Table 2). Images of untreated sickle cell blood and blood samples treated with methanolic extracts of Ipocrata, SolMon, Cari-oil and VerMyg are shown in Figures 1, 2, 3, 4 and 5, respectively.

### Effect of plant methanolic extract-treatment on G6PDH activity, Fe^2+^/Fe^3+^ ratios and hemoglobin concentration

The G6PDH activity in HbSSM was significantly higher (P < 0.05) compared with NBM and NBF samples (Table 1). The Fe^2+^/Fe^3+^ ratios in HbSSM were significantly reduced (P < 0.05) by all the plant extract-treatments compared with control (HbSSM without extract) (Table 3). Hemoglobin concentration was significantly increased (P < 0.05) in HbSSM groups treated with any of the plant extracts compared with VerMyg (Table 3). However, hemoglobin concentrations were only significantly increased (P < 0.05) by SolMon in HbSSF compared with VerMyg. There was no significant effect (P > 0.05) of the plant extract-treatment on Hb/Met-Hb ratios in either HbSSM or HbSSF blood.

**Table 3:** The effect of selected plant extracts on the chemical properties of sickle cell blood

**Treatment**	**Fe**^**2+**^**/Fe**^**3+**^**Ratio**		**Hemoglobin concentration (%)**		**Hb/Met-Hb ratio (× 10**^**-1**^**)**		**Sickle cell polymerization inhibition (× 10**^**-4**^**) (Unit/min)**	
	**Male**	**Female**	**Male**	**Female**	**Male**	**Female**	**Male**	**Female**
Control	9.94 ± 5.07	5.34 ± 1.40	NT	NT	NT	NT	8.50 ± 7.00	0.00 ± 0.00
*Vernonia amygdalina*	4.49 ± 1.29	3.66 ± 1.53	15.34 ± 7.34	29.52 ± 16.01	0.91 ± 0.47	4.59 ± 3.44	16.43 ± 4.12	21.50 ± 6.61
*Ipomoea involucrata*	3.45 ± 1.19	2.78 ± 1.82	34.25 ± 8.59	51.99 ± 26.89	1.01 ± 0.47	2.69 ± 1.85	6.00 ± 4.29	18.50 ± 10.12
*Solenostemon monostachyus*	2.33 ± 0.49	1.48 ± 0.33	43.16 ± 17.51	132.78 ± 97.61	0.88 ± 0.31	1.85 ± 0.49	41.33 ± 9.28	94.00 ± 17.28
*Carica papaya* seed oil	4.52 ± 1.83	3.00 ± 0.96	25.33 ± 8.37	34.22 ± 18.26	1.44 ± 0.84	2.72 ± 1.74	14.50 ± 9.69	15.50 ± 6.27

Data expressed as mean ± SD (*n* = 10). Hb = hemoglobin; Met-Hb= methemoglobin; NT = not tested.

### Effect of plant methanolic extract-treatment on LDH activity and inhibition of sickle cell polymerization

Inhibition of sickle cell polymerization in HbSSF was significantly increased (P < 0.05) by all the plant extract-treatments studied, compared with the control (HbSSF treated with saline solution). However, SolMon treatment alone significantly inhibited (P < 0.05) sickle cell polymerization in HbSSM compared with the controls (Table 3). In HbSSF, sickle cell polymerization inhibition by SolMon plant extract was significantly higher (P < 0.05) compared with VerMyg treatment. However, in HbSSM, sickle cell polymerization inhibition was significantly higher (P < 0.05) in the SolMon and Ipocrata-treated groups compared with that of VerMyg (Table 3). All the plant extracts significantly reduced (P < 0.05) LDH activity in HbSSM and HbSSF treated blood (Table 4).

**Table 4:** The effect of methanolic plant extracts on lactate dehydrogenase activity of human sickle cell blood

**Treatment**	**Lactate dehydrogenase activity (U/L)**	
	**Male**	**Female**
Control	204.70 ± 35.09	123.81 ± 37.97
*Vernonia amygdalina*	9.90 ± 6.26	12.13 ± 4.48
*Ipomoea involucrate*	6.95 ± 1.01	3.90 ± 3.79
*Solenostemon monostachyus*	6.19 ± 5.07	4.23 ± 2.42
*Carica papaya* seed oil	152.70 ± 45.77	14.03 ± 8.56

Data expressed as mean ± SD (*n* = 5).

### Effect of plant methanolic extract-treatment on blood catalase and peroxidase activity

Higher catalase activity was found in NBF than that of the NBM (Table 5). All plant extract treatments, including glutathione, significantly reduced (P < 0.05) catalase activity in NBF compared with the control (NBF without extract) (Table 5). However, Cari-oil, VerMyg and Ipocrata treated NBF had significantly higher (P < 0.05) catalase activity compared with the glutathione standard (Table 5). In NBM, Cari-oil, Ipocrata, and glutathione significantly reduced (P < 0.05) the catalase activity compared with the control (NBM without extract) (Table 5), whereas VerMyg extract significantly increased (P < 0.05) the catalase activity in NBM compared with the control (NBM without extract). Furthermore, the SolMon, Cari-oil and VerMyg treated NBM had significantly higher (P < 0.05) catalase activity compared with the glutathione group (Table 5). Interestingly, no significant effect (P > 0.05) of glutathione or plant extract treatments were observed on catalase activity in HbSSM compared with the control (HbSSM without extract) (Table 5). In HbSSF, only glutathione significantly reduced (P < 0.05) the catalase activity compared with the control (HbSSF without extract), although the catalase activity was significantly increased in HbSSF treated with Ipocrata compared with glutathione (Table 5).

**Table 5:** Catalase activity in plant extract-treated sickle cell and healthy blood

**Treatment**	**Catalase activity (μmol H**_**2**_**O**_**2**_**/min)**			
	**Healthy blood donor**		**Sickle cell blood**	
	**Male**	**Female**	**Male**	**Female**
Control	46.40 ± 6.37	152.53 ± 46.10	9.24 ± 4.44	36.27 ± 16.11
Glutathione	5.33 ± 4.44	8.0 ± 8.95	3.20 ± 2.75	8.00 ± 7.47
*Vernonia amygdalina*	69.69 ± 10.08	29.87 ± 2.13	44.09 ± 12.94	22.16 ± 6.90
*Ipomoea involucrata*	8.53 ± 0.00	38.40 ± 11.27	19.91 ± 9.619	22.04 ± 2.46
*Solenostemon monostachyus*	34.13 ± 13.32	13.87 ± 3.70	14.93 ± 5.64	15.64 ± 3.26
*Carica papaya* seed oil	14.93 ± 5.64	40.53 ± 14.93	25.60 ± 10.67	25.60 ± 8.11

Data expressed as mean ± SD (*n* = 10).

Both Cari-oil and Ipocrata significantly increased (P < 0.05) peroxidase activity in NBM and NBF compared with their respective control (blood without extract) (Table 6). Similar significant increases (P < 0.05) in peroxidase activity by Cari-oil and Ipocrata treatment were also observed in NBM and NBF compared with glutathione treatment (Table 6). Only SolMon treatment significantly reduced (P < 0.05) the peroxidase activity in both NBM and NBF compared with glutathione (Table 6). Cari-oil treated HbSSF had significantly increased (P < 0.05) peroxidase activity compared with both the control (HbSSF without extract) and glutathione groups (Table 6), but in HbSSM it only induced a significant increase (P < 0.05) in peroxidase activity when compared with control (HbSSM without extract) (Table 6). There was no significant effect (P > 0.05) on peroxidase activity in HbSSM treated with any of the plant extracts when compared with the glutathione group.

**Table 6:** Peroxidase activity in plant extract-treated sickle cell and healthy blood

**Treatment**	**Peroxidase activity (× 10**^**-5**^**Unit/sec)**			
	**Healthy blood donor**		**Sickle cell blood**	
	**Male**	**Female**	**Male**	**Female**
Control	5.46 ± 4.00	3.46 ± 2.00	2.89 ± 1.60	3.83 ± 1.93
Glutathione	8.07 ± 1.10	6.37 ± 0.49	4.76 ± 2.89	5.83 ± 1.10
*Vernonia amygdalina*	10.00 ± 0.00	8.00 ± 0.09	3.62 ± 1.90	2.47 ± 1.60
*Ipomoea involucrata*	18.06 ± 2.40	15.16 ± 3.11	3.74 ± 1.02	7.49 ± 2.90
*Solenostemon monostachyus*	2.09 ± 0.60	3.19 ± 0.77	2.14 ± 0.20	3.43 ± 1.90
*Carica papaya* seed oil	62.30 ± 18.00	57.30 ± 11.30	17.24 ± 6.90	19.85 ± 5.40

Data expressed as mean ± SD (*n* = 10).

## Discussion

The discovery of alternate or synergistic therapy for the treatment of SCD due to increasing concerns regarding its effect on the health of individuals worldwide is gaining attention. Higher PCV was observed in healthy male blood compared with healthy female blood (Table 1). This confirmed the findings of an earlier report [18,19]. The mean PCV values in normal healthy individuals range from 42 to 45% in adult males, 36-48% in adult females, and 32 to 65% in children. Values lower than these indicate an abnormality [20]. Lower PCV values observed in HbSSM and HbSSF compared with NBM and NBF may be attributed to the high rate of hemolysis in HbSS blood [21] that leads to a shortened life span (15.2 ± 6.3 days) of sickle cell blood compared with normal blood (90-120 days) [22]. Similar to the reduction in sickle blood cell counts induced by VerMyg (2.42 ± 1.11%), Cari-oil, SolMon and Ipocrata also effectively reduced the numbers of sickle blood cells in the untreated sickle cell patients’ blood (26.85 ± 3.12%) to that of 1.03 ± 0.16, 1.90 ± 0.43 and 4.04 ± 0.71% respectively. All plant extract treatments had greater antisickling efficacy compared with previous reports using extracts from the antisickling plant (VerMyg). However, these extracts did not completely reverse sickling since sickle blood cells still existed in HbSSM and HbSSF blood compared with normal healthy controls (Table 2). Treatment with SolMon, Ipocrata, and Cari-oil extracts had similar antisickling properties to VerMyg since higher normal RBC levels were observed in the treated sickle blood compared with untreated sickle blood. This is evident in the images of Ipocrata (Figure 2), SolMon (Figure 3) and Cari-oil (Figure 4) treated HbSS blood, where fewer numbers of sickle blood cells were observed compared with the large amount of normal RBCs. However, Ipocrata treatment was less effective compared with VerMyg treatment as measured by the number of percentage of sickle cells present in the blood sample (Table 2). In this study, three plants (SolMon, Ipomea and Cari-oil) were shown to have novel antisickling properties. The antisickling property of Cari-oil was expected since the antisickling properties of *C*. *papaya* fruits was previously reported [15]. This study also demonstrated that the seed oil from *C*. *papaya* also possesses antisickling properties. The levels of aggregation were reduced in all the plant extract-treated HbSS blood compared with that of untreated HbSS blood (Figures 1, 2, 3, 4 and 5).

The high prevalence of HbSS diseases and G6PDH deficiency (A^-^ type) in tropical areas is related to the selective advantage these diseases provide against malaria [23]. The protective effect of HbSS against malaria has been attributed to heme oxygenase-1 (HO-1), an enzyme whose expression is strongly induced by sickle hemoglobin [24]. G6PDH deficiency screening among SCD patients has provided the opportunity to administer appropriate preventive and therapeutic measures [25]. G6PDH activity in HbSSM was higher compared with either healthy male or female donor blood (Table 1), suggesting that male HbSS patients may be more susceptible to malaria than female HbSS patients may. A higher prevalence of malaria was reported in males than in females [26-28]. Symptomatic patients are almost exclusively male, due to the X-linked pattern of inheritance [29]. Therefore, G6PDH deficiency may be more prevalent in HbSS females than in HbSS males, and suggests why a higher occurrence of SCD-associated hemolytic crises (anemia, pain, jaundice) often occurs in female HbSS patients [30].

An increase in Fe^2+/^Fe^3+^ratios upon application of a drug or plant extract indicates a reversal of sickling, suggesting conversion of deoxyHbS to oxyHbS [31]. Fe^2+/^Fe^3+^ ratios were reduced by all methanolic plant extract treatments in HbSS males (Table 3), suggesting they may decrease the oxygen affinity of RBCs in HbSS male patients [31]. All plant extract treatments generally led to an increase in hemoglobin concentrations in HbSS female patients (Table 3). Interestingly, SolMon and Ipocrata treatments were more effective than VerMyg for increasing hemoglobin levels in HbSSM. However, only SolMon treatment was more effective than VerMyg in HbSSF.

One particular area of focus in the management of SCDs is the inhibition of sickle cell hemoglobin polymerization. It was hypothesized that an ideal antisickling drug or agent should significantly inhibit polymerization of the sickle cell hemoglobin in SCD blood [32]. Apart from inhibiting polymerization, the agent should also increase the oxygen affinity of hemoglobin. In this study, polymerization was inhibited by VerMyg plant extract treatment in male SCD patients (Table 3). This was expected as the plant had previously been reported to have antisickling properties [14]. Similarly, SolMon exhibited a remarkable ability to inhibit sickle cell polymerization in male SCD patients (Table 3). Of note, all plant extracts tested in this study inhibited sickle cell polymerization in HbSSF. Importantly, the potency of SolMon to inhibit sickle cell hemoglobin polymerization exceeded that of VerMyg in HbSSF as previously described (Table 3).

### The effect of plant extracts on oxidative stress in Sickle cell patient bloods

#### Lactate dehydrogenase

LDH is an important enzyme for carbohydrate metabolism and is used as an indicative criterion for exposure to chemical stress [33]. All plant extracts in this study reduced LDH activity in sickle cell blood (Table 4). Reduced LDH activity levels in treated HbSS blood samples (Table 4) indicate a lower energy metabolism of RBCs [34]. In addition, all plant extracts facilitated biochemical changes that enhanced oxidative interactions in HbSS blood, indicating anaerobic fermentation in RBCs [33]. Therefore, the plants studied here possess the ability to alleviate stress associated with HbSS patients. Hence, the plant extract treatment may enhance HbSS blood stability and patient health. This study also revealed that the antisickling properties of these plants might function by modulating the genes responsible for LDH synthesis in both sexes. The wide variation of LDH activities between both sexes is expected as indicated in the control. Genetic differences between both sexes had been linked to the wide variation [35]. This wide variation in LDH activity between both sexes was similarly manifested only in the samples treated with Cari-oil (Table 4). This is obviously due to the fact that the seed oil could only generate a mild effect in modulating the genes that coded for LDH in male. The same seed oil also generated drastic effect in modulating the genes that code for LDH in female subjects. This insignificant variation in LDH activity in males as against a remarkable variation in females had also been reported [36].

#### Catalase

The catalase enzyme is an endogenous antioxidant present in all aerobic cells and facilitates the removal of toxic hydrogen peroxide by catalyzing its decomposition to molecular oxygen and water, without the production of free radicals [37]. It is therefore useful as a defense agent against oxidative stress [37]. High amounts of free radicals are important in the development of SCD complications and other disease states [38]. High catalase activity also suggests poor health status of the blood [38]. Catalase activity is induced by the presence of its substrate, hydrogen peroxide. Thus, a strong antioxidant should reduce catalase activity as shown by glutathione treatment (Table 5).

The low catalase activity in NBM compared to that of the NBF in this work is similar to earlier reports [39,40]. These difference was attributed to be due to aging since aging decreases catalase gene transcription in male while in female, aging leads to an increase in the translational efficiency of catalase mRNA [41]. Glutathione and the Ipocrata, VerMyg, Cari-oil and SolMon plant extracts reduced the catalase activity in NBF. However, the Cari-oil, VerMyg, and Ipocrata extracts were less effective than glutathione. Although Cari-oil, Ipocrata and glutathione reduced catalase activity in NBM, VerMyg reduced the antioxidant status of male blood by increasing its catalase activity. The antioxidant potential of glutathione was more effective than SolMon in NBM (Table 5).

However, Ipocrata, VerMyg, Cari-oil and SolMon plant extracts had no significant effect on the catalase activity of either male or female sickle cell blood (Table 5). The fact that the plant extracts were effective in healthy blood indicates that higher hydrogen peroxide substrate concentrations than were used in this study (0.5% w/v), may be required for plant extracts to be effective in reducing catalase activity in sickle cell blood [42]. Alternatively, catalase inhibitors such as β-mercaptoethanol and dithiothreitol may be abundantly present in HbSS patients. These inhibitors possess the ability to modify cysteine residues in the catalase enzyme. Some catalase inhibitors are thiol compounds that can also modify the heme group of catalase, and may suggest the cause of enzyme inactivation in sickle cell patient blood [43].

#### Peroxidase activity

Peroxidase activity normally increases in response to increased substrate (hydrogen peroxide) concentrations in the blood [44]. However, only SolMon exhibited effective antioxidant activity by reducing the peroxidase enzyme levels in NBM and NBF (Table 6). Cari-oil and Ipocrata treatment of NBM and NBF blood may encourage free radical generation (H_2_O_2_) since treatment increased peroxidase activity (Table 6). Similar free radical generation effects were also observed for the Cari-oil extract-treated HbSSM and HbSSM blood (Table 6).

## Conclusions

The positive effects exhibited by all plant extracts in reducing LDH activity were independent of the sex of HbSS patients. However, the effects on other biochemical indices (enzymes) considered in this study were sex dependent. While all plant extracts were effective in enhancing HbSS polymerization inhibition in females, only SolMon and Ipocrata were effective in male HbSS blood. In addition, all the plant extracts may be harmful to SCD patients’ blood since they increased catalase activity in HbSSM blood, whereas only Ipomoea may be harmful to SCD patients’ blood since it increased catalase activity in HbSSF blood. Furthermore, Cari-oil positively modulated peroxidase activity in both HbSS sexes, whereas only SoleMon positively modulated peroxidase in male HbSS blood. Thus, the effectiveness of these plants in managing SCD is generally dependent on the sex of the HbSS patients, and this should be considered when administering plant extracts for the management of SCD.

Future studies should isolate and study the bioactive compounds responsible for the antisickling potential in these plants. Anthocyanins have recently been implicated in the antisickling properties of plants [5]. The methanolic extract of *S*. *monostachyus* and *I*. *involucrata* leaves, and that of *C*. *papaya* seed oil had individual advantages relative to each other, which were manifest in their antisickling properties. The combination of these plant extracts may be employed for the management of SCD to take advantage of their possible synergistic effects.

## Abbreviations

VerMyg: *Vernonia amygdalina*; SoleMon: *Solenostemon monostachyus*; Ipocrata: *Ipomoea involucrata*; Cari-Oil: *Carica papaya* seed oil; SCD: Sickle-cell disease; HbSS: Human sickle cell blood; HbSSM: Female human sickle cell blood; HbSSM: Male human sickle cell blood; NBM: Healthy male human blood; NBF: Healthy female human blood; RBC: Red blood cell; G6PDH: Glucose-6-phosphate dehydrogenase; LDH: Lactate dehydrogenase; PCV: Packed cell volume.

## Competing interests

The authors declare that they have no competing interests.

## Authors’ contributions

ISA discovered the potential of plant extracts for curing sickle cell, designed the research plan, performed statistical analysis, interpreted the results and prepared the manuscript. IOO was involved in sickle cell blood collection, and analyzed the G6PDH activity, Fe^2+^/Fe^3+^ ratios, and polymerization inhibition. ODF was involved in sickle cell blood collection from patients, and analyzed the catalase and peroxidase activities. PIU was involved in blood collection from healthy male donors, and performed analysis of sickling reversal activity, sickle cell and red blood cell counts and sample slide preparation. DOO was involved in blood collection from healthy female donors. TBA performed the LDH activity analysis. AOA performed the PCV determination. BTA performed operation of the microscope and photographing of the blood samples. All authors read and approved the final manuscript

## Authors’ information

ISA currently is a Lecturer, as well as the Biochemistry Program Coordinator since joining Covenant University, Ota, Ogun State, Nigeria on 21^st^ October 2008. He previously served as Assistant Quality Assurance Manager at GlaxoSmithKline Consumer Nigeria PLC (2007-2008). ISA rose to the position of Senior Research Officer during 10 years of research working experience at the Nigerian Stored Products Research Institute, Ilorin, Kwara State, Nigeria (1997-2007). He has also worked in many capacities, as a teacher under the National Youth Service Corps at Government Secondary School, Gwandu, Kebbi State-Nigeria (1994-1995) and as a Research/Marketing officer at Blafy International Associate, Ikoyi-Lagos (1996-1997). He obtained his Ph.D. degree (Biochemistry) in 2008, having obtained a Master of Science degree in 2001 and Bachelor of Science degree in 1994 in the same field (Biochemistry) at the University of Ilorin, Ilorin, Nigeria. In addition, he was awarded a Postgraduate Diploma in Computer Science in 2002 from the University of Ilorin. He also obtained a Diploma in Marketing at the University of Lagos, Akoka-Lagos in 1995, and a Proficiency certificate in Computer Science in 1995 at the French Cultural Centre, Ikoyi, Lagos, Nigeria. He was awarded a Certificate of Fellowship in 2003 by The United Nations University, Tokyo, Japan after success with his advanced research/training in Food Science and Technology carried out at the Central Food Technological Research Institute, Mysore-India. His major research contribution involves:

1. Reducing national food waste through the optimization of the drying performance of a developed hybrid dryer that uses both kerosene and solar radiation for energy generation.

2. Development of an edible bio-wax (Bemul-wax) for preservation of agro-crops.

3. Developing methods for using the developed bio-wax for storing sweet oranges for a minimum period of four months without loss of its aesthetic, nutritional and sensory values.

He is currently working on adding value to certain under-utilized plants and agro-waste (*Carica papaya* seed) by researching into possible ways of utilizing them, and their potential for improving human health.

## Pre-publication history

The pre-publication history for this paper can be accessed here:

http://www.biomedcentral.com/1472-6882/12/262/prepub
